# Explicit and Implicit Approach vs. Avoidance Tendencies towards High vs. Low Calorie Food Cues in Patients with Obesity and Active Binge Eating Disorder

**DOI:** 10.3390/nu9101068

**Published:** 2017-09-27

**Authors:** Georgios Paslakis, Simone Kühn, Sebastian Grunert, Yesim Erim

**Affiliations:** 1 Department of Psychosomatic Medicine and Psychotherapy, University Hospital Erlangen, Schwabachanlage 6, 91054 Erlangen, Germany; sebastian.grunert@me.com (S.G.); yesim.erim@uk-erlangen.de (Y.E.); 2 University Clinic Hamburg-Eppendorf, Clinic and Policlinic for Psychiatry and Psychotherapy, Martinistraße 52, 20246 Hamburg, Germany; kuehn@mpib-berlin.mpg.de

**Keywords:** approach avoidance task (AAT), binge eating disorder, explicit, implicit, psychotherapy, training

## Abstract

Patients with binge eating disorder (BED) suffer from regular food binges with loss of control. This may be due to dysfunctional approach vs. avoidance tendencies towards food in BED. We applied an approach-avoidance task (AAT), in which *n* = 24 patients with obesity and active BED (OB-BED), *n* = 32 patients with obesity without current BED (OB), and *n* = 25 healthy controls (CO) either approached (“pulled”) or avoided (“pushed”) high (HC) vs. low calorie (LC) food pictures. We tested the hypothesis that OB-BED patients would show an approach bias (measured as different response times RT) towards HC food compared to the other groups. While there was no main effect for group or direction of movement, a significant main effect for calorie (*p* < 0.001; RT for HC significantly slower than for LC) was found. Repeated measures ANOVA (rm-ANOVA) for comparison of OB-BED vs. OB vs. CO revealed a significant three-fold interaction group × direction × calorie (*p* = 0.02). Against our hypothesis, the OB-BED group showed an avoidance bias for LC. In explicit ratings, OB-BED reported a significantly reduced urge to consume LC food compared to the OB group. Similar to OB-BED, CO also showed an avoidance bias for LC. The implications of our results are discussed and future directions in this field of research are presented.

## 1. Introduction

Binge eating disorder (BED) is a common eating disorder affecting approximately 2% of the global population [1]. Patients suffering from BED are characterized by frequent disruptions in their eating patterns leading to regular binge episodes, at least once a week for three months. A binge episode in the context of BED is defined as eating an objectively large amount of food in a certain amount of time accompanied with the feeling of loss of behavioral control. Binges cause feelings of disgust, guilt, and regret [2]. In contrast to bulimia nervosa, patients with BED do not display compensatory behaviors (e.g., purging or the use of laxatives). As a result, BED is often associated with obesity; the prevalence of BED may be as high as 30–57% among individuals with obesity [3,4]. The DSM-V criteria [2] for BED are displayed in Table 1. The occurrence of regular binge episodes suggests an altered valuation of food in patients with BED compared to individuals without this eating disorder. Understanding the underlying pathomechanisms of BED is important, in order to be able to design adequate interventions in the battle against the disorder.

Evolutionary reasoning suggests that, in order to adapt to environmental conditions and to survive, organisms need to approach rewards and avoid punishment [5]. In this sense, approach behavior is defined as behavior directed towards positive stimuli, whereas avoidance behavior is defined as behavior directed away from negative stimuli (e.g., [6,7,8]). The basic compatibility effect between positive vs. negative valence and action tendencies of approach vs. avoidance, respectively, has been replicated many times with different types of apparatus, with different reference frames (e.g., self vs. object), and with a broad range of affective stimuli [9].

Food is a rewarding stimulus, as it activates central structures of the brain’s reward system, e.g., the ventral striatum. The visual presentation of food stimuli vs. pictures of neutral objects (e.g., pieces of furniture) distinctly activates brain structures involved in reward processing [10,11]. Such activation patterns are significantly more pronounced for high-calorie, compared to low-calorie food [12]. The response to reward is underpinned by the incentive valence of cues that is often referred to as “wanting”. Usually, the higher the incentive valence of a certain food stimulus is, the more pronounced is the approach bias towards it [13] and the higher is the likelihood of its consumption [14,15,16]. Activation in rewarding brain circuits in response to food stimuli have been found to be higher in individuals with obesity compared to normal-weight controls [17]. Hence, altered rewarding properties of food could predispose the occurrence of binge episodes, particularly while facing an abundance of high palatable, high-calorie food [18,19]. Among individuals with obesity, those with BED form a distinct subgroup with a greater level of functional impairment [20]. A series of studies have investigated inhibitory control, mental flexibility, decision-making, attention to stimuli related to body, and food and brain activation patterns in response to food stimuli or other rewarding stimuli (e.g., monetary rewards) in patients with BED using a variety of methods, such as fMRI, event-related potentials and other psychophysiological measures of the sympathetic and parasympathetic response system and neuropsychological paradigms (e.g., Stroop and dot probe paradigm, Iowa Gambling Task, customized mental flexibility tasks, etc.). These studies have shown that patients suffering from BED show behavioral and cognitive abnormalities in evaluation of rewards and losses, executive functions, attentional bias towards food, and impulsivity [21,22,23,24,25].

On the psychological level, the mechanisms of approach vs. avoidance of food-intake stand under the influence of both conscious (explicit) and automatic (implicit) regulatory processes. Bargh’s automotive theory of non-conscious goal pursuit [26] postulates that specific stimuli are able to induce certain approach or avoidance behaviors automatically and in the absence of consciousness, provided that a strong interconnection between the stimulus and the target behavior has occurred in the past. Approach-avoidance behaviors subsume all responses ranging from those that are entirely automatic to those being entirely under conscious control and a clear distinction is not easy. Only approach and avoidance behaviors to which the individual has conscious access can be assessed by means of clinical interviews. Likewise, subjective ratings in questionnaires may mirror explicit motives, but they are liable to confounding factors (e.g., social desirability) and are therefore not capable of reliably assessing altered (e.g., desensitized) motivational driving forces. Self-reports that are supplemented by indirect measures assessing automatic, uncontrolled implicit processes are, therefore, at an advantage [27]. Reaction times, e.g., constitute such indirect (implicit) measures and are highly useful add-ons to the explicit approach. Systematic differences in reaction times assessed in experimental paradigms measuring implicit preferences may, for instance, allow for inferences on cognition and attitudes that have an influence on behaviors, but are not accessible to the conscious self. This kind of associative/implicit cognitive processes might be relevant for the onset and perpetuation of eating disorders.

The approach-avoidance task (AAT) has been developed [28] as an experimental paradigm that tests associative processes that are not influenced by attention, memory, interpretation, or strategic control [29]. The AAT is a behavioral reaction time task that assesses approach and avoidance motivational processes by instructing participants to respond to cues (pictures) by either “pulling” these towards themselves or “pushing” them away, while responding to an irrelevant feature of the stimulus (e.g., the presented picture format as landscape vs. portrait, and not according to the picture content itself). The amount of time required to initiate these actions is the dependent variable. Such responses can be captured by means of motor movements of the forearm; unpleasant cues are pushed away from oneself faster; pleasant cues are pulled faster—interfering with the explicit instruction to respond to an irrelevant feature of the stimulus. Thus, a close connection between the valence of stimuli and the herewith-associated motor reaction exists. The AAT uses this connection, in order to assess the association between stimuli and behavioral responses.

The literature attests validity in measuring approach-avoidance motivational processes to the AAT [30,31]. In the context of anxiety and addiction research it has been shown that positively-connoted cues were associated with an approach, and negative cues with an avoidance bias [32]. In a previous investigation, our group has shown that healthy controls displayed an approach bias for high- and low-calorie food cues; this bias was absent in the group of anorexia nervosa (AN) patients [33]. In a non-clinical cohort, Brockmeyer et al. [34] applied a food approach-avoidance task and could show that high food cravers displayed a stronger automatic approach bias towards food than low food cravers. Using self-reports and indirect (via facial electromyography) valuation of food vs. non-food cues in patients with obesity and BED compared to subjects with obesity without BED, Leehr et al. [35] found a diverging self-reported (positive) and indirect (negative) valuation of food stimuli in patients with obesity and BED.

The present study is the first to implement an AAT for the assessment of implicit bias towards high- vs. low-calorie food stimuli in a sample of patients suffering from BED. In contrast to the Implicit Association Test (IAT) or the Affective Priming Task, the AAT does not capture congruence effects between two stimuli or the affects which are evoked by two stimuli, but the congruence effect between a visual stimulus and a behavioral reaction in terms of a movement (here: approach/pull vs. avoid/push). The AAT is a different test inasmuch as participants are asked to either pull cues towards them in a flexing arm movement or push cues away by extending their arm. Thus, a more authentic subjective perception of approach vs. avoidance may be achieved. Our study cohort includes patients with obesity and BED (OB-BED), subjects with obesity without current BED (OB), and a normal-weight healthy control group (CO). We tested the hypothesis that a significantly greater approach bias for high-calorie stimuli (measured as significantly different response times (RTs)) would be found in OB-BED patients compared to the other groups. Assuming that there would be no differences between the groups, explicit preferences by means of self-ratings of the presented cues were also assessed.

## 2. Materials and Methods

### 2.1. Participants

A total of *n* = 24 patients with an active BED (OB-BED) were included in the present study at the Department of Psychosomatic Medicine and Psychotherapy of the University Hospital in Erlangen, Germany. For the diagnosis of BED, the DSM-V criteria of BED had to be fulfilled [2]. BED was ascertained during non-standardized clinical interviews carried out by psychologists and physicians with long experience in the treatment of eating disorders. OB-BED patients had sought outpatient consultation in our department for their eating behavior and/or underwent a psychosomatic appraisal procedure before bariatric surgery. In addition, inpatients who were admitted to the ward for treatment of their BED were included in the study. Patients received inpatient behavioral therapy eating disorder treatment for eight weeks. This treatment includes supervised regular meal intake and other behavioral treatment interventions, e.g., psychoeducation, behavioral analyses, motivational interventions to promote behavioral change, promotion of self-efficacy, creation of explanatory models of the disorder, body and food cue exposure, emotion regulation techniques (“skills”), relapse prevention strategies, etc. Dieting is not part of this program and weight loss is not the primary goal of treatment. Furthermore, *n* = 32 subjects (OB) who sought outpatient consultation/appraisal before bariatric surgery and were not given a BED diagnosis, as well as inpatients seeking treatment for conditions other than an eating disorder (e.g., depression) were included in the study. 

In order to keep phenotypes as homogenous as possible, we included in the analyses only subjects (with or without acute BED) with a body mass index (BMI) ≥ 30 kg/m^2^ and norm-weight controls with a BMI > 18.5 kg/m^2^, but below ≤ 25 kg/m^2^. In some cases, OB subjects did report objective binge eating episodes, but did not fulfill the DSM-V criteria of BED, e.g., with regard to the frequency of binge eating. Thus, apart from subjects without a lifetime diagnosis of BED, the OB group comprised subclinical binge eaters as well. In addition, the OB group also comprised patients with a lifetime diagnosis of BED who reported a period of more than 30 days without even a single objective binge eating episode with loss of control and were therefore not considered as having a current (“acute”) BED. Similarly, inpatients receiving treatment for BED who had been more than four weeks in inpatient treatment and had not suffered even a single binge eating episode during this time were also allocated to the OB group.

The group of *n* = 25 healthy controls with absent self-reported lifetime BED consisted of members of all professions (e.g., nurses, laboratory staff, medical professionals) at the University Hospital of Erlangen, as well as medical students, who were not familiar with the test procedure and were randomly asked to participate. All healthy controls were asked if they suffered binge episodes in the past or at present, and were only enrolled in case they did not suffer from an eating disorder; however, a thorough diagnostic interview for lifetime eating disorders was not performed. All healthy controls were also asked if they suffered purging following meals and if they used laxatives or diuretics for weight control. Healthy controls were deemed healthy according to their BMI and their scores in eating disorder-specific questionnaires.

The study had been approved by the local ethics committee. All subjects were over 18 years of age. Participants were weighed before the test procedure for calculation of BMI using self-reported height. Only participants whose last meal had taken place four hours (or less) before the test procedure were included, in order to ensure comparability in terms of satiety.

### 2.2. Test Procedure

**Figure 1:**
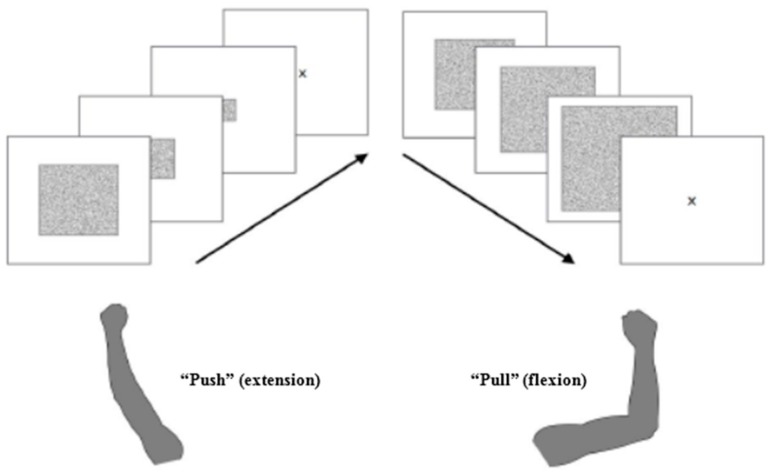
Participants were instructed to either “pull” or “push” food pictures shown on a computer screen based on the presented picture format (landscape vs. portrait format) by moving the computer mouse towards or away from themselves. “Pulling” a picture (flexion of the forearm) made it larger until it filled almost the complete screen and then disappeared, while “pushing” a food picture (forearm extension) caused the picture to shrink to a dot and disappear from the screen (for more details see main text).

All participants were instructed to either “pull” or “push” food pictures shown on a computer screen by moving the computer mouse towards or away from themselves (instead of using a joystick as in similar previous studies). Participants were instructed to “push” or “pull” based on the presented picture format (landscape vs. portrait format). The specific cue format was not associated with a specific required response (e.g., “pull” for all cues in landscape format and “push” for all cues in portrait format). There were two versions of the test: in the first version, participants were asked to “pull” cues in portrait format, while in the parallel version, participants were asked to “push” cues in portrait format. Thus, response mapping was counterbalanced between participants in a 1:1 manner. Motor responses in both directions caused the picture size to change: “pulling” a picture made it larger until it almost filled the entire screen and then disappeared, while “pushing” a food picture caused the picture to shrink to a dot and disappear from the screen (Figure 1). This visual feedback elicits the strong subjective impression that participants actually approach (“pull”) or avoid (“push”) food (pictures). As this was a reaction time paradigm, participants were instructed to react as fast as possible, but still as accurately as they can. At the beginning of each trial, a fixation cross was presented. The trial was initiated by the participant by clicking on the fixation cross using the computer mouse. In this paradigm, 50% of the cues contained a high- and 50% contained a low-calorie food cue. Each of the stimuli was presented both in landscape as well as portrait format in random order. Before the actual experimental run, participants underwent a practice run, in which they learned to “push” or “pull” *n* = 20 white rectangles, otherwise following the same instructions as described above. While a visual feedback was given in case of an erroneous motor response during the practice run, false motor responses during the experimental run were excluded from RT analyses. All participants used their dominant hand. Participants did not complete any other tests before taking part in this study. The AAT took place at different times during the day; the earliest AAT was performed at 10.00 a.m. and no AAT was performed after 16.00 p.m.

### 2.3. Stimuli

Forty pictures were selected from the food.pics database [36], twenty pictures displaying high-calorie foods (HC) and twenty pictures displaying low-calorie foods (LC). The pictures from this database all have the same resolution and color depth and are homogenous with regard to background and camera distance. The database delivers information on calorie content and physical features of the food pictures (e.g., visual complexity). High-calorie cues included foods like a hamburger and French fries, a piece of cream cake, pizza, ice cream, or a plate of spaghetti with tomato sauce, while low-calorie cues consisted of items like green salad, apples, sweet peppers, a cucumber, crispbread with curd cheese, etc. The following cues from the food.pics database [36] were used: numbers 0003, 0004, 0010, 0016, 0028, 0032, 0043, 0046, 0048, 0067, 0096, 0110, 0116, 0131, 0143, 0153, 0166, 0173, 0176, and 0189 for HC food cues, and 0194, 0198, 0200, 0202, 0203, 0205, 0208, 0215, 0216, 0219, 0227, 0232, 0234, 0238, 0241, 0254, 0256, 0267, 0284, and 0280 for LC food cues. Food items of the two categories differed significantly in kcal per 100 g (HC: 706.10 ± 750.17 vs. LC: 89.85 ± 77.38 kcal; *p* = 0.002).

### 2.4. Anthropometric and Self-Report Measures

A series of variables such as time since last meal (in hours), age, weight and height (allowing the calculation of BMI), and medication (number of prescribed drugs including psychotropic drugs) were assessed. The presence of depression (“yes”/”no”) was based on clinical judgment (interview).

All participants were asked to fill in the Eating Disorder Examination-Questionnaire (EDE-Q) and the Eating Disorder Inventory-2 (EDI-2). The EDE-Q [37] evaluates eating disorder psychopathology in the past 28 days. It contains 22 items that are answered on a seven-point scale ranging from “never” to “every day”. It comprises four subscales assessing (a) restraint; (b) eating concern; (c) weight concern; and (d) shape concern. Internal consistency of the German version was α = 0.97 in a validation study [38]. As this study included inpatients whose meal intake—according to a multidisciplinary inpatient behavioral therapy treatment program—was monitored by caregivers (in order to ensure regular food intake), the subscale “restraint” was not applicable in this study. Thus, the EDE-Q total score was calculated based on the three remaining subscales. The EDI-2 [39] is a self-report questionnaire with 91 items that assesses eating disorder psychopathology, but also inter- and intrapersonal aspects thought to be relevant for the development and perpetuation of eating disorders [39] (e.g., perfectionism, interpersonal distrust, lack of interoceptive awareness, etc.). Higher scores indicate more severe psychopathology; only the total score of the EDI-2 was considered in the analyses.

### 2.5. Explicit Ratings

Following the experimental run, participants were asked to rate the presented high- and low-calorie pictures on a scale ranging from 0 to 100 with regard to the following questions: (a) How much would you like to eat this food right now? (urge to eat); (b) How much would you regret having eating this food? (regret); and (c) how healthy is this food in your opinion? (healthiness). Since the “urge” to consume the presented food (cues) was assessed, it was thought to be important to also assess “regret” doing so. Ratings of healthiness were included, in order to examine if individuals with obesity with/without BED consider HC or LC foods more or less “healthy” compared to norm-weight healthy controls.

### 2.6. Data Analysis

Data analysis was carried out using MATLAB (The MathWorks, Inc., Natick, MA, USA) and the Statistical Package for the Social Sciences (SPSS 21; Armonk, NY, USA: IBM Corp.). Only correct motor responses during the experimental run were included in RT analyses. RT data were tested for normal distribution. Repeated-measures analyses of variance (rm-ANOVA) were carried out for between-group analyses. In fact, two repeated measure factors were defined: (a) “calorie” with regard to high-calorie vs. low-calorie food cue content and; (b) “direction” of motion with regard to “push” vs. “pull”. For within-group analyses, univariate and multivariate analyses of variance (ANOVA, MANOVA) were performed as appropriate. Unless otherwise mentioned, the Games-Howell test was chosen for subsequent post hoc analyses (due to unequal variances). *p* ≤ 0.05 was set as level of significance. RTs are reported as median ± standard deviation. Median response times were included in the analyses due to lower sensitivity to outliers compared with mean scores [28,31,40,41]. Accordingly, no low or high RT thresholds and no RT outliers were defined; all recorded RTs were included in the analyses. Anthropometric variables (age, BMI etc.), scores in self-report questionnaires and subjective ratings of food pictures are reported as mean ± standard deviation.

## 3. Results

Patients with obesity and BED (OB-BED) vs. subjects with obesity (OB) vs. norm-weight controls (CO).

The three groups did not differ significantly with regard to sex (OB-BED: 20 females and four males vs. OB: 22 females and 10 males vs. CO: 18 females and seven males; F_2,78_ = 0.79, *p* = 0.46), age (OB-BED: 40.54 ± 11.69 vs. OB: 44.78 ± 10.07 vs. CO: 39.36 ± 11.23 years.; F_2,78_ = 1.97, *p* = 0.15), and time since last meal (OB-BED: 1.94 ± 0.71 vs. OB: 2.06 ± 1.06 vs. CO: 1.72 ± 0.93 hrs.; F_2,78_ = 0.83, *p* = 0.39). However, as expected, the three groups differed significantly in their BMI (OB-BED: 44.04 ± 10.45 vs. OB: 43.71 ± 9.43 vs. 22.29 ± 1.61 kg/m^2^; F_2,78_ = 58.80, *p* < 0.001; post hoc (Games-Howell): OB-BED > CO and OB > CO, *p* < 0.001), as well as regarding all subscores and total scores in the applied eating disorder-specific questionnaires (EDE-Q and EDI-2) (Table 2).

In addition, the three groups differed significantly with regard to frequency of diagnosed depression (F_2,72_ = 15.94, *p* < 0.001; post hoc (Games-Howell): OB-BED > CO and OB > CO, *p* < 0.001). Therefore, depression was entered as a factor in subsequent RT analyses (rm-ANOVA; see below). 

Self-reported mean duration of illness in the BED group was 22.5 ± 14.79 years.

### 3.1. Explicit Ratings

The OB-BED group differed from the OB group only in significantly lower ratings of their “urge” to consume low-calorie food pictures (*p* = 0.04), but not in ratings of “regret” or “healthiness” of the presented cues. Compared to CO, both the OB-BED and the OB group showed higher ratings of “regret” for high-calorie food cues, but OB reported lower ratings of “regret” for low-calorie food cues compared to the CO group (Table 3). In addition, the OB-BED and the OB group rated “healthiness” of HC food cues significantly lower than the CO group. However, the OB group rated “healthiness” of LC food cues significantly higher than the CO group (Table 3).

### 3.2. Errors

The OB-BED and the OB group did not differ with regard to errors in all conditions under examination. The OB-BED and the CO groups differed significantly with regard to errors in all conditions under examination (push/HC, pull/HC, push/LC, pull/LC). Errors and error frequencies in % per condition (push/pull and HC/LC) × group are shown in Table 4.

### 3.3. Response Time (RT)

Response time (RT; in milliseconds) was defined as the sum of the onset of the first motor response in reaction to the stimulus and the length of the motor movement and was selected as the dependent variable. Trials in which errors were made (e.g., initiating “pull” on a “push” trial, and vice versa) were excluded from the analysis. Between-group analyses were obtained by means of a repeated-measures analysis of variance (rm-ANOVA) with “calorie” (HC: high-calorie vs. LC: low-calorie) and “direction” of motion (push vs. pull) being the two repeated-measures factors.

The rm-ANOVA with RTs as dependent variables and “group” (here: OB-BED vs. OB vs. CO) and “depression” as between-subject factors, revealed no main effect for “group” (F_2,70_ = 2.85; *p* = 0.07) or “depression” (F_1,70_ = 0.34; *p* = 0.56). There was also no main effect for “direction” of motion (F_1,70_ = 0.03; *p* = 0.87). However, there was a main effect for “calorie” content (F_1,70_ = 24.69; *p* < 0.001). Thus, RTs were significantly different depending on whether the presented cues were high calorie (slower RTs) or low calorie (faster RTs). Interestingly, a significant interaction “group” × “calorie” × “direction” (OB-BED/OB/CO × HC/LC × push/pull) (F_2,70_ = 4.02; *p* = 0.02) was found, indicating that the groups had significantly different RTs with regard to “calorie” and “direction” of motion. 

RTs did not significantly differ between the OB-BED and the OB group in none of the push/HC, pull/HC, push/LC or pull/LC conditions (Table 5). With the exception of push/HC, the OB group showed significantly slower RTs in all other conditions compared to the CO group (Table 5). Table 5 shows details on response times (RTs) per investigated group.

**Figure 2:**
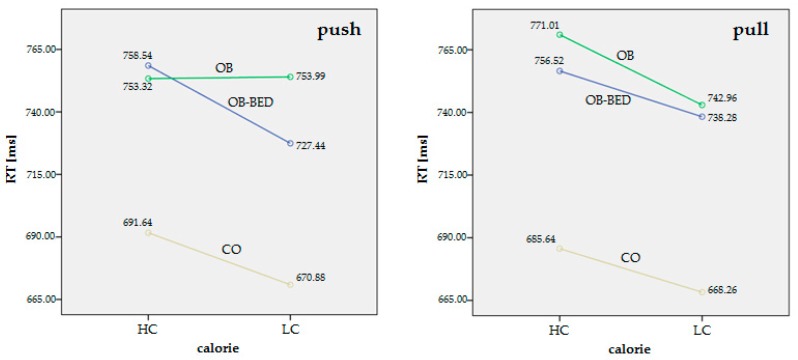
This figure shows the results of rm-ANOVA with two repeated measures factors (calorie: high-calorie (HC) vs. low-calorie (LC) food cue content and direction of motion: push vs. pull). As a main result, a three-fold interaction “group” × “calorie” × “direction” (OB-BED/OB/CO × HC/LC × push/pull) was found (F_2,70_ = 4.02; *p* = 0.02). Within the groups, RTs may be interpreted as an avoidance bias for LC in the OB-BED group and an approach bias for LC in the OB group (for more details see main text). OB-BED = patients with obesity and current BED, OB = subjects with obesity without BED, CO = norm-weight controls without self-reported lifetime eating disorder diagnosis. HC = high-calorie food picture, LC = low-calorie food picture. RT = reaction time in milliseconds (ms).

Within the groups, patients with obesity and BED (OB-BED) showed significantly faster response times (RTs) for push/LC compared to push/HC (*p* = 0.01) (Figure 2); this may be interpreted as an avoidance bias for LC food cues. This bias was present in the group of CO (*p* = 0.003), but absent in the group of subjects with obesity without BED (OB), in which RTs for push/LC were practically identical to those for push/HC (Figure 2). In contrast, the OB group showed faster RTs for pull/LC compared to pull/HC (*p* = 0.001), pointing towards an approach bias for LC food cues. Again, this bias was present in the group of CO (*p* = 0.01), but absent in the group of OB-BED (Figure 2).

## 4. Discussion

Binge eating disorder (BED) is characterized by episodes of binge eating occurring at least once a week for three months and associated with a lack of control over eating and absence of compensatory behaviors [2]. The occurrence of regular binge eating episodes suggests that the valuation process of food stimuli in BED might be different from individuals without BED. Studies analyzing the processing of food stimuli in individuals with eating disorders (for comprehensive reviews see [42,43,44]) have predominantly investigated anorexia and bulimia nervosa, while studies on BED are scarce. Initial brain imaging, eye tracking data, and behavioral test paradigms indicate that, in response to food stimuli, patients with BED show a higher arousal rate, a concurrent motor plan to start eating, a higher sensitivity to reinforcement, a higher reward sensitivity, and greater inhibitory deficits as compared to individuals without BED [23,45,46,47,48,49].

As a main result, a three-fold interaction “group” × “calorie” × “direction” (OB-BED/OB/CO × HC/LC × push/pull) was found. The OB-BED group showed an avoidance bias for LC food cues, expressed by significantly faster response times (RTs) for push/LC compared to push/HC. This bias was absent in the group of OB subjects, in which RTs for push/LC were practically identical to those for push/HC. In contrast, the OB group showed an approach bias for LC, mirrored by faster RTs for pull/LC compared to pull/HC. This bias was absent in the group of OB-BED. The results in explicit ratings, in which the OB-BED group reported a significantly lower urge to consume LC food (pictures), corresponds well to the implicit findings as found by the AAT. Interestingly, the two groups (OB-BED vs. OB) could not be distinguished according to the eating disorder-specific self-report questionnaires EDE-Q and EDI-2. 

As the second main result, on the implicit level, we found that healthy controls also showed an avoidance bias for LC food cues, similar to the OB-BED group. There is no obvious explanation for this rather surprising result. However, as we assessed implicit bias independent of binge eating episodes, this result gives reason to believe that enduring implicit preferences, as measured by the AAT, may not be suitable to explain the acute occurrence of the “binge”-phenotype. Considering that binge eating episodes occur in certain affectively-loaded situations (e.g., sadness, anger, etc.), the next plausible step would be to examine the influence of mood induction upon implicit preferences towards food cues. Maybe then differences between OB-BED and controls—that were not detected in the present study—could be unmasked. Additionally, healthy controls did not differ in their explicit ratings of the urge to consume HC or LC food compared to the OB-BED group. 

We also found that the calorie content of the food pictures had an impact on RTs in all investigated groups (high-calorie stimuli resulted in slower RTs and low-calorie stimuli resulted in faster RTs), independent of the direction of motion. This slowing in response could reflect that high-calorie food was interfering with the ability to respond quickly in all groups. Interestingly, OB-BED patients performed significantly more errors compared to CO (in both directions push/pull and for both calorie contents HC/LC), while there were no significant differences in performed errors between the OB-BED and the OB group. Global RTs were significantly slower in the OB group compared to CO. A study using the AAT with neutral objects vs. food cues could help shed light on the question, whether global slower RTs are stimulus-specific or if other factors should be made accountable (e.g., impairments in forearm reactions due to body size). Finally, “healthiness” of HC food cues was underrated in both the OB-BED and the OB compared to the CO group; on the other hand, the OB group considered “healthiness” of LC food cues to be higher compared to the CO group.

Binge eating and obesity have been independently associated with attentional bias to food stimuli [50,51,52]. An attentional bias towards food has been found in several studies in populations with obesity leading to the assumption that attentional bias towards food may be relevant to the development and maintenance of obesity [53,54,55,56,57]. Individuals with obesity and concurrent binge eating also have increased attentional bias to food compared to individuals with obesity and to normal-weight controls [25,46,58,59,60]. Thus, our result of an avoidance bias for LC food cues in patients with obesity and BED is not in line with previous studies.

However, not all studies using indirect measures of a bias towards food in patients with BED have proven a clear approach bias. In a comparison of food versus non-food stimuli, Leehr et al. [35] examined self-reported and indirect (via facial electromyography) valuation in an overweight sample with BED, an overweight sample without BED, and normal-weight controls. The BED sample reported a significantly more positive food bias compared to the overweight sample without BED. However, indirect valuation of food stimuli was negative in all groups. Svaldi et al. (2010), examined patients with BED and overweight controls who were exposed to high versus low calorie food stimuli and also found higher negative valuation (as assessed by means of event related potentials) for high calorie foods in both groups [25].

This study presents data of an AAT in BED. To our knowledge, this is the first application of an AAT, in order to assess implicit preferences in obese patients with current BED (OB-BED) and compared them to implicit preferences found in subjects with obesity without current BED (OB). In addition, while previous patient studies on different disorders using the AAT have applied disorder-specific cues (e.g., alcohol or spiders) against neutral cues (e.g., neutral objects), in the present investigation, a more subtle cue differentiation into high- and low-calorie food pictures has been utilized. Imaging studies have shown that high- and low-calorie food cues are processed in the brain differently [61,62,63].

Methodological differences among various studies may also account for differences in observed attentional bias to food in individuals with obesity [24,55,64]. Task sensitivity might be affected by the type of stimuli used (e.g., words or pictures), task parameters, such as stimulus duration [55], participants’ hedonic mindset, or being on a weight-reducing diet [54]. In a newly-published study by Deluchi et al., participants with obesity and BED vs. participants with obesity, but without BED performed a computerized task designed to evaluate attentional bias towards food in different stages of the attentional process. Both groups showed positive attentional bias to food in the initial orientation stage (100 ms), whereas bias was almost absent in the maintenance of attention stage (2000 ms), suggesting ambivalent attentional responses to food stimuli. When displayed for 500 ms, only the group with BED showed a bias towards food images; thus, disengaging from food-related stimuli was slower in the BED group [65].

A strength of the present study is the inclusion of a control group and the use of both implicit and explicit measures. Our results are to be considered specific, as patients with OB-BED and the group of OB did not differ in their overall response times (independent of calorie content or direction of motion). Moreover, we have attempted to examine an acute BED phenotype (patients actively engaging in objective binge eating episodes) against subjects with obesity without current BED. Distinguishing between such groups is a strenuous endeavor due to overlapping phenotypes; e.g., patients with obesity may suffer from objective binge eating episodes, but not as often, as to be classified as patients with BED, and, on the other hand, there are patients diagnosed with BED in the past, but not currently engaging in binge eating behavior. These difficulties actually reflect a fundamental problem: the efforts of classification in diagnostic categories that are yet insufficiently circumscribed. Setting the cut-off at one month was self-determined and somewhat arbitrary; binge episodes that had occurred longer than four weeks ago were not considered to mirror “acute” symptom occurrence. Finally, because all participants were exposed to the same set of stimuli, it would also be useful to apply individually selected stimuli of preferred food (cues) in future studies.

A limitation of the present study lies especially within its cross-sectional design, as approach-avoidance tendencies are states, rather than traits and might change over time. Our group of patients with obesity and BED consisted, in part, of patients who were receiving inpatient treatment for their BED. This might have influenced the results, as it has been described that individuals who were in treatment for eating disorders, including BED, showed reduced attentional bias towards food-related stimuli [66]. Attentional bias towards food-related stimuli was reduced after weight loss due to bariatric surgery at a six-month follow-up in the study by Giel et al. [67]. Thus, studying only untreated patients with BED would be required, in order to be able to allocate experimental observations to specific cohort characteristics. As a further limitation, the socio-economic status of participants was not explicitly assessed. 

While implicit processes may not be consistent with current explicit goals, such as maintaining a low-calorie diet [68], they may be important in future cognitive behavioral strategies [69,70]. Current cognitive behavioral therapy aims at conscious, explicit, deliberate processes, while implicit, more automatic processes outside of awareness or control and activated by associative clusters may not be not adequately addressed by main behavioral treatment interventions [71]. The effectiveness of implicit treatments using the principles of the AAT is yet to be proven in the context of eating disorders. Some studies show promising results, but the overall evidence base is rather scarce. In a study of subclinical bulimic eating disorder psychopathology, participants were trained to make avoidance movements in response to visual food stimuli in ten sessions (each lasted 15 minutes) over a five-week course; the approach bias towards high-calorie food shown at baseline was significantly reduced and turned into an avoidance bias after the training [34]. Recently, it was shown that approach bias modification reduces the approach bias towards food cues and actual food intake in female student populations [72,73]. As shown in a previous study by our group, implicit AAT training might make sense in the case of female patients with anorexia nervosa, in an attempt to increase the approach bias of food in general [33]. New interventions targeting implicit processes in terms of a bias “retraining” may prove to be useful strategies against obesity as well [54,74]. However, due to overlapping phenotypes, it remains unclear which patients may benefit the most from such interventions.

## Figures and Tables

**Table 1:** DSM-V diagnostic criteria for binge eating disorder [2].

Recurrent episodes of binge eating. An episode of binge eating is characterized by both of the following: eating, in a discrete period of time, an amount of food that is definitely larger than most people would eat in a similar period of time under similar circumstances a sense of lack of control over eating during the episode
The binge-eating episodes are associated with three (or more) of the following: eating much more rapidly than normal eating until feeling uncomfortably full eating large amounts of food when not feeling physically hungry eating alone because of feeling embarrassed by how much one is eating feeling disgusted with oneself, depressed, or very guilty afterwards
Marked distress regarding binge eating is present
The binge eating occurs, on average, at least once a week for three months
The binge eating is not associated with the recurrent use of inappropriate compensatory behavior (e.g., purging) and does not occur exclusively during the course anorexia nervosa, bulimia nervosa, or avoidant/restrictive food intake disorder

**Table 2:** Comparisons (MANOVA) between OB-BED, OB, and CO with regard to self-reports using the EDE-Q and the EDI-2 (total scores and subscales). Meal intake of inpatients in this study was monitored by caregivers during inpatient treatment; thus, the subscale “restraint” of the EDE-Q was not applicable (for more details see main text). OB-BED = patients with obesity and current BED, OB = subjects with obesity without binge eating episodes, CO = norm-weight controls without self-reported lifetime eating disorder diagnosis.

	OB-BED	OB	CO	F (*p*)	Post Hoc
EDE-Q	3.67 ± 1.23	3.56 ± 1.57	0.59 ± 0.55	F_2,78_ = 52.02 (*p* < 0.001) **	OB-BED > CO and OB > CO, *p* < 0.001 **
EDE-Q shape concern	4.46 ± 1.13	4.18 ± 1.58	0.91 ± 0.77	F_2,78_ = 64.66 (*p* < 0.001) **	OB-BED > CO and OB > CO, *p* < 0.001 **
EDE-Q weight concern	3.87 ± 1.18	3.76 ± 1.50	0.65 ± 0.76	F_2,78_ = 58.39 (*p* < 0.001) **	OB-BED > CO and OB > CO, *p* < 0.001 **
EDE-Q eating concern	2.61 ± 1.70	2.37 ± 1.85	0.22 ± 0.35	F_2,78_ = 19.69 (*p* < 0.001) **	OB-BED > CO and OB > CO, *p* < 0.001 **
EDI-2	315.04 ± 69.75	309.66 ± 63.82	180.68 ± 29.65	F_2,78_ = 44.87 (*p* < 0.001) **	OB-BED > CO and OB > CO, *p* < 0.001 **

** Indicate a < 0.001 level of significance.

**Table 3:** Comparisons (MANOVA) between OB-BED, OB and CO with regard to explicit ratings; all participants were asked the following questions: (a) how much would you like to eat this food right now? (urge to eat); (b) how much would you regret eating this food? (regret); and (c) how healthy is this food in your opinion? (healthiness). OB-BED = patients with obesity and current BED, OB = subjects with obesity without binge eating episodes, CO = norm-weight controls without self-reported lifetime eating disorder diagnosis. HC = high-calorie food picture, LC = low-calorie food picture.

	OB-BED	OB	CO	F (*p*)	Post Hoc
rating urge HC	55.61 ± 19.10	55.08 ± 13.32	58.74 ± 14.90	F_2,76_ = 0.42 (*p* = 0.66)	--
rating regret HC	71.61 ± 20.18	72.85 ± 15.80	49.21 ± 17.45	F_2,76_ = 14.64 (*p* < 0.001) **	OB-BED > CO and OB > CO, *p* ≤ 0.001 **
rating healthiness HC	11.28 ± 6.42	10.21 ± 4.46	16.55 ± 5.93	F_2,76_ = 9.82 (***p* < 0.001**) **	OB-BED < CO and OB < CO, *p* ≤ 0.01 *
rating urge LC	68.60 ± 18.69	79.82 ± 10.18	76.63 ± 9.30	F_2,76_ = 5.03 (*p* = 0.01) *	OB-BED < OB, *p* = 0.04 *
rating regret LC	10.76 ± 11.77	5.87 ± 6.07	12.59 ± 12.64	F_2,76_ = 3.25 (*p* = 0.04) *	OB < CO, *p* = 0.05 *
rating healthiness LC	91.67 ± 8.20	95.42 ± 4.03	90.88 ± 4.70	F_2,76_ = 5.07 (*p* = 0.01) *	OB > CO, *p* = 0.001 **

* and ** indicate a ≤ 0.05 and < 0.001 level of significance, respectively.

**Table 4:** Comparisons (MANOVA) between OB-BED, OB, and CO with regard to errors per condition (push/pull and HC/LC) x group. Error frequencies in % are shown in brackets. OB-BED = patients with obesity and current BED, OB = subjects with obesity without BED, CO = norm-weight controls without self-reported lifetime eating disorder diagnosis. HC = high-calorie food picture, LC = low-calorie food picture.

	OB-BED *n* = 24	OB *n* = 32	CO *n* = 25	F (*p*)	Post Hoc
Errors push/HC: (% total trials)	11.58 ± 7.90 (14.06)	9.38 ± 6.68 (11.80)	5.88 ± 4.06 (7.35)	F_2.78_ = 4.95 (*p* = 0.01) *	OB-BED > CO and OB > CO, *p* ≤ 0.05 *
Errors pull/HC: (% total trials)	11.88 ± 8.51 (14.84)	8.91 ± 6.37 (11.13)	6.44 ± 4.07 (8.15)	F_2.78_ = 4.25 (*p* = 0.02) *	OB-BED > CO, *p* = 0.02 *
Errors push/LC: (% total trials)	10.96 ± 7.63 (13.70)	9.31 ± 6.77 (11.64)	6.20 ± 3.46 (7.75)	F_2.78_ = 3.70 (*p* = 0.03) *	OB-BED > CO, *p* = 0.02 *
Errors pull/LC: (% total trials)	11.96 ± 6.99 (14.95)	8.94 ± 6.30 (11.17)	6.52 ± 3.57 (8.15)	F_2.78_ = 5.33 (*p* = 0.01) *	OB-BED > CO, *p* = 0.01 *

* Indicates a ≤ 0.05 level of significance.

**Table 5:** Median values, standard deviation (SD) and range (minimum – maximum) per condition (push/pull vs. HC/LC) × group for response times (RTs) (in ms). A MANOVA was performed for comparisons between the groups. OB-BED = patients with obesity and current BED, OB = subjects with obesity without BED, CO = norm-weight controls without self-reported lifetime eating disorder diagnosis. HC = high-calorie food picture, LC = low-calorie food picture.

	OB-BED *n* = 24	OB *n* = 32	CO *n* = 25	F (*p*)	Post Hoc
RT push/HC: median ± SD (range)	751.40 ± 129.02 (550.00–1051.00)	751.78 ± 130.58 (508.00–1067.00)	691.64 ± 118.09 (517.00–905.00)	F_2.78_ = 1.95 (*p* = 0.15)	--
RT pull/HC: median ± SD (range)	756.06 ± 143.51 (502.50–1058.50)	767.77 ± 121.17 (565.50–1070.00)	685.64 ± 107.24 (521.00–862.00)	F_2.78_ = 3.39 (*p* = 0.04) *	OB > CO, *p* = 0.02 *
RT push/LC: median ± SD (range)	720.42 ± 127.54 (503.00–1038.00)	750.75 ± 126.79 (537.00–991.00)	670.88 ± 108.28 (518.00–838.00)	F_2.78_ = 3.04 (*p* = 0.05) *	OB > CO, *p* = 0.04 *
RT pull/LC: median ± SD (range)	744.08 ± 151.44 (489.50–1067.00)	741.84 ± 119.91 (542.50–1021.00)	668.26 ± 101.74 (503.00–847.50)	F_2.78_ = 3.07 (*p* = 0.05) *	OB > CO, *p* = 0.04 *

* Indicates a ≤ 0.05 level of significance.
